# The Role of Blood-Based Biomarkers in Transforming Alzheimer’s Disease Research and Clinical Management: A Review

**DOI:** 10.3390/ijms26178564

**Published:** 2025-09-03

**Authors:** Vera Pacoova Dal Maschio, Fausto Roveta, Lucrezia Bonino, Silvia Boschi, Innocenzo Rainero, Elisa Rubino

**Affiliations:** 1 Department of Neuroscience “Rita Levi-Montalcini”, University of Turin, 10126 Turin, Italy; vera.pacoovadalmaschio@unito.it (V.P.D.M.); fausto.roveta@unito.it (F.R.); lucrezia.bonino@unito.it (L.B.); silvia.boschi@unito.it (S.B.); innocenzo.rainero@unito.it (I.R.); 2 Center for Cognitive Disorders and Dementias (CDCD), Department of Neuroscience and Mental Health, Azienda Ospedaliera Universitaria Città della Salute e della Scienza di Torino, 10126 Turin, Italy

**Keywords:** Alzheimer’s disease, blood biomarkers, pathophysiology, GFAP, p-Tau217

## Abstract

Alzheimer’s disease (AD) is a progressive neurodegenerative condition representing the most common cause of dementia and currently affects millions of people worldwide. The clinical presentation includes memory impairment, cognitive decline, and neuropsychiatric symptoms, reflecting pathological hallmarks such as β-amyloid (Aβ) plaques, neurofibrillary tangles, synaptic dysfunction, and neuroinflammation. Despite being the gold standard for detecting amyloid and tau pathologies in vivo, cerebrospinal fluid (CSF) biomarkers and positron emission tomography (PET) imaging are not widely used in the clinical setting because of invasiveness, high costs, and restricted accessibility. Recent advances in blood-based biomarkers offer a promising and minimally invasive tool for early detection, diagnosis, and monitoring of AD. Ultra-sensitive analytical platforms, including single-molecule arrays (Simoa) and immunoprecipitation-mass spectrometry, now enable reliable quantification of plasma Aβ isoforms, phosphorylated tau variants (p-Tau181, p-Tau217, p-Tau231), neurofilament light chain (NfL) and glial fibrillary acidic protein (GFAP). In addition, blood biomarkers reflecting oxidative stress, neuroinflammation, synaptic disruption and metabolic dysfunction are under active investigation. This narrative review synthesizes current evidence on blood-based biomarkers in AD, emphasizing their biological relevance, diagnostic accuracy, and clinical applications. Finally, we highlight forthcoming challenges, such as standardization, and future directions, including the use of artificial intelligence in precision medicine.

## 1. Introduction

Alzheimer’s disease (AD) is a complex neurodegenerative disorder, already impacting millions worldwide, and it is anticipated to reach markedly higher prevalence by mid-century due to global demographic shifts. Accounting for 60–70% of dementia cases, AD is marked by progressive cognitive and functional decline, with patients experiencing memory loss, impaired executive functions, behavioral changes, and, ultimately, a complete loss of independence [1]. The societal and economic burden of AD is profound, impacting globally not only patients, but also caregivers, healthcare systems and economies [2].

The pathophysiology of AD is driven by the accumulation of β-amyloid (Aβ) plaques and neurofibrillary tangles composed of hyperphosphorylated tau (p-Tau) protein [3]. These aggregates disrupt neuronal communication and trigger neuronal loss, particularly in brain regions critical for memory and cognition, such as the medial temporal lobe and more specifically the hippocampus [4]. Notably, these pathological changes begin years, often decades, before the onset of clinical symptoms, creating a lengthy preclinical phase that presents both a challenge and an opportunity for early intervention [5].

Traditionally, AD diagnosis has relied on clinical assessment, supplemented by confirmatory tests such as cerebrospinal fluid (CSF) analysis and positron emission tomography (PET) imaging to detect amyloid and tau pathology [6]. Even if these methods are accurate and well established, their high costs, invasiveness, and limited accessibility restrict their routinary use, especially in primary care and large-scale screening.

A major advancement in the field has been the introduction of the ATN classification framework by the National Institute on Aging and Alzheimer’s Association (NIA-AA), which organizes biomarkers into three categories: amyloid deposition (A), tau pathology (T), and neurodegeneration or neuronal injury (N) [6]. This framework has enhanced diagnostic precision and deepened our understanding of AD biology. Initially reliant on CSF and imaging biomarkers, the framework is now increasingly supported by evidence from plasma-based biomarkers, which are showing comparable accuracy [7].

In 2024, the ATN framework underwent a significant revision, reflecting the rapid evolution of biomarker research and the growing clinical adoption of less invasive diagnostic tools [7]. The updated ATN criteria have been designed to be more inclusive and flexible, explicitly recognizing the validity of both fluid and imaging biomarkers, and, for the first time, formally incorporating blood-based biomarkers into the diagnostic algorithm. This change is a direct response to mounting evidence that plasma assays for Aβ isoforms, p-Tau species (such as p-Tau181 and p-Tau217), and neurofilament light chain (NfL) can reliably mirror the pathological processes previously assessed only through cerebrospinal fluid analysis or advanced neuroimaging techniques.

The revised ATN system maintains its tripartite structure but now allows clinicians and researchers to select the most appropriate and accessible biomarker modalities for each category, depending on available resources, patient characteristics, and the clinical context. This approach is suitable in healthcare settings and aims to democratize access to biological diagnosis of AD, especially in regions where lumbar puncture or PET imaging may not be feasible [8].

Another important conceptual advance in the 2024 revision is the reaffirmation that the ATN framework is fundamentally a biological, rather than a syndromic, classification. The presence or absence of amyloid, tau, and neurodegeneration biomarkers is used to define the biological state of AD, irrespective of clinical symptoms. This approach supports the recognition of a continuum that spans from preclinical, asymptomatic individuals with biomarker evidence of AD pathology, to those with mild cognitive impairment and, ultimately, dementia [9]. By decoupling the biological diagnosis from the clinical syndrome, the revised ATN criteria could facilitate an earlier identification of at-risk individuals, a more precise stratification for clinical trials and allow an intervention before irreversible neurodegeneration occurs [10].

Furthermore, the 2024 update provides clearer guidance on the interpretation of discordant biomarker profiles and highlights the importance of longitudinal assessment, recognizing that individuals may transition between ATN categories over time. The framework also underscores the growing role of ATN status in guiding therapeutic decisions, particularly with the advent of disease-modifying treatments that target specific pathological processes. As such, the revised criteria are expected to accelerate the ongoing integration of blood-based biomarkers into routine clinical practice, paving the way for more accessible, scalable, and personalized approaches to the diagnosis and management of AD [11]. Indeed, updated guidelines consistently encourage the evolution of daily clinical practice, prompting clinicians to adopt new protocols, and overcoming practical obstacles.

Blood-based biomarkers have emerged as a transformative alternative to current diagnostic procedures, offering a minimally invasive, accessible, and cost-effective solution for screening, early detection, and longitudinal monitoring [12,13]. Technological innovations, including ultrasensitive immunoassays, mass spectrometry, single-molecule arrays (Simoa), and automated platforms such as Lumipulse, now enable the detection of low-abundance biomarkers, such as Aβ isoforms, p-Tau, and NfL in blood with high sensitivity and specificity [14].

The advent of monoclonal antibody therapies targeting amyloid plaques, such as lecanemab and donanemab, has further underscored the importance of early diagnosis, as their therapeutic efficacy depends on identifying suitable patients in the earliest disease stages [15]. Blood-based biomarkers are thus poised to play a crucial role in patient selection, monitoring therapeutic response, and advancing personalized medicine in AD [16].

This narrative review provides a comprehensive overview of the current landscape of blood biomarkers in AD, examining their biological relevance and diagnostic utility. We also address challenges related to standardization, cross-platform variability, population diversity, and ethical considerations, and outline future directions for the integration of blood-based biomarkers into clinical and research practice.

## 2. Blood Biomarkers in Alzheimer’s Disease

### 2.1. Amyloid-β

Aβ peptides, particularly Aβ40 and the more aggregation-prone Aβ42, are central to AD pathogenesis and the foundation of the amyloid cascade hypothesis [17]. These peptides are generated from the amyloid precursor protein (APP) through sequential cleavage by β-secretase (BACE1) and γ-secretase [18]. An imbalance between Aβ production and clearance leads to the oligomerization and deposition of Aβ42 into extracellular plaques, which disrupt neuronal communication, promote oxidative stress, activate microglia, and ultimately contribute to synaptic dysfunction and neuronal death [3]. Notably, Aβ accumulation begins up to two decades before clinical symptoms appear, making it one of the earliest detectable events in AD [19].

Traditionally, the ratio of Aβ42/40 in cerebrospinal fluid has served as a reliable biomarker of cerebral amyloid burden, correlating strongly with amyloid PET imaging [7]. However, CSF collection via lumbar puncture is relatively invasive and impractical for large-scale screening or routine clinical use. This is particularly true in elderly patients, who represent the primary population affected by AD. In this group, lumbar puncture poses additional challenges due to age-related physiological changes such as decreased cerebrospinal fluid flow and altered CSF composition, which can complicate sample collection and interpretation. Moreover, elderly patients often have comorbidities, frailty, and spinal degenerative changes that increase the risk of procedural complications and patient discomfort [20]. The procedure may also be contraindicated in individuals taking anticoagulants or those with bleeding disorders, as the risk of spinal hematoma is elevated. These factors collectively limit the feasibility and safety of lumbar puncture in the elderly, underscoring the urgent need for minimally invasive alternatives.

The development of blood-based assays for Aβ has thus been a major goal, offering a minimally invasive and accessible alternative.

Historically, detecting Aβ in plasma was challenging due to its low abundance, peripheral metabolism, and binding to carrier proteins, such as albumin and lipoproteins, which limited both sensitivity and specificity [21]. Recent technological advances have transformed this landscape. Immunoprecipitation-mass spectrometry (IP-MS) and single molecule array platforms now enable the sensitive and specific quantification of plasma Aβ42/Aβ40 ratios [22]. Large cohort studies, including ADNI, BioFINDER, and AIBL, have demonstrated that plasma Aβ42/40 correlate with amyloid PET status, supporting its use as a non-invasive diagnostic tool [23]. Studies have demonstrated that plasma Aβ42/40 measured via IP-MS could distinguish amyloid-positive individuals with an area under the curve (AUC) exceeding 0.88, and could predict amyloid pathology up to eight years before clinical onset [24,25].

AUC is the key indicator of diagnostic accuracy in a Receiver Operator Characteristic (ROC) analysis conducted to compare different diagnostic tests [26]. The AUC value reflects the accuracy of a test in distinguishing two populations (e.g., healthy controls and patients), and it ranges from 0.50, when population differentiation is random, to 1, when the two populations are perfectly recognized and separated by the studied test [26]. Therefore, an AUC of 0.88, as demonstrated by plasma Aβ42/40, reflects a reliable diagnostic accuracy.

Despite these advances, several challenges remain. Plasma Aβ levels are influenced by biological variability, including renal function, vascular pathology, systemic inflammation, and APOE-ε4 genotype [27]. The dynamic range of Aβ changes in plasma is also relatively narrow, which may limit sensitivity for population screening or early disease detection compared to healthy controls. Consequently, plasma Aβ42/40 is most effective when used as part of a multi-biomarker panel rather than as a stand-alone diagnostic hallmark [28].

Currently, plasma Aβ42/40 could be utilized in clinical trials for participant pre-screening, risk stratification, and as a gatekeeper to more expensive confirmatory testing such as PET or CSF analysis. Its ability to detect amyloid changes years before symptom onset underlines its value for preclinical screening and identifying candidates for preventive interventions [19]. Ongoing efforts to harmonize assays, establish robust clinical cut-offs, and account for confounding factors, are essential for the widespread adoption of plasma Aβ biomarkers in both research and clinical practice.

### 2.2. Tau Proteins

Tau proteins are integral to neuronal function, playing a vital role in stabilizing microtubules and maintaining axonal integrity, which are in turn essential for intracellular transport and neuronal communication [29]. In AD, tau undergoes pathological hyperphosphorylation, a process that causes it to detach from microtubules, misfold, and aggregate into intracellular neurofibrillary tangles (NFTs) [30]. These NFTs are a defining neuropathological hallmark of AD and are closely linked to synaptic dysfunction and cognitive decline [4]. The progression of tau pathology follows a characteristic spatiotemporal pattern, initially affecting the entorhinal cortex before spreading to the hippocampus and neocortex, a sequence described by Braak staging [31]. This predictable pattern correlates with the clinical progression of cognitive symptoms, underscoring the central role of Tau in disease evolution.

In recent years, phosphorylated tau isoforms detectable in blood have emerged as valuable biomarkers for AD diagnosis, disease monitoring, and therapeutic evaluation [32]. As for many other biomarkers, blood levels of phosphorylated Tau isoforms should be integrated in a comprehensive approach that also includes clinical evaluation, neuroimaging, and other laboratory tests. Given that plasma p-Tau can be elevated in other neurological and neuromuscular conditions [33], such a multidimensional protocol is essential to ensure an accurate diagnosis and avoid misclassification. Indeed, a recent study highlights that p-Tau increases are not exclusive to AD pathology, but can also occur in amyotrophic lateral sclerosis (ALS) and certain myopathies [33]. This overlap underscores the potential for false-positive results if plasma p-Tau levels are interpreted alone. Consequently, neurological examination, detailed clinical history and complementary investigations remain indispensable components of the diagnostic process.

Among phosphorylated tau isoforms, p-Tau181 has been one of the most extensively studied. Plasma levels of p-Tau181 show strong correlations with cerebrospinal fluid p-Tau181 and tau positron emission tomography imaging, reflecting central nervous system tau pathology [34]. Importantly, p-Tau181 levels begin to rise early in the disease continuum, including during the preclinical phase, when cognitive symptoms are not yet evident. This early increase allows p-Tau181 to discriminate AD from other neurodegenerative dementias, such as frontotemporal dementia (FTD), with a diagnostic accuracy often reflected by AUC values exceeding 0.85 [35,36]. Furthermore, longitudinal studies have demonstrated that rising plasma p-Tau181 levels predict cognitive decline and the progression from mild cognitive impairment (MCI) to overt dementia, highlighting its prognostic value [37].

More recently, p-Tau217 has also garnered considerable attention, due to its superior specificity and diagnostic accuracy compared to p-Tau181 [38]. This isoform more effectively distinguishes AD from other tauopathies, including progressive supranuclear palsy (PSP) and frontotemporal dementia (FTD) [39]. For instance, certain studies have shown that plasma p-Tau217 correlates more closely with both amyloid and tau PET imaging than several CSF biomarkers, achieving an AUC above 0.90 in differentiating AD from controls [40,41]. The heightened specificity of p-Tau217 has led to its increasing adoption in clinical trials, particularly those targeting amyloid pathology, such as the TRAILBLAZER-ALZ study, where it is used both for participant selection and therapeutic response monitoring [42,43].

Another promising biomarker, p-Tau231, is gaining recognition as an ultra-early indicator of AD pathology. Unlike p-Tau181 and p-Tau217, p-Tau231 levels rise even before tau PET imaging becomes positive, reflecting the earliest molecular changes induced by amyloid accumulation and tau misprocessing [44,45]. Emerging evidence suggests that plasma p-Tau231 can predict cognitive decline and amyloid PET positivity during prodromal stages of AD, underscoring its potential utility for very early diagnosis and intervention [46]. Supporting this, a recent head-to-head study comparing plasma and cerebrospinal fluid p-Tau217, p-Tau181, and p-Tau231 in a memory clinic cohort demonstrated that while plasma p-Tau217 showed the highest overall diagnostic accuracy, plasma p-Tau231 was particularly sensitive in identifying early amyloid pathology, highlighting its value as a biomarker for the earliest stages of AD progression [47].

These phosphorylated tau biomarkers are increasingly integrated into clinical research, not only as inclusion criteria for trials, but also as surrogate endpoints to track disease progression and assess therapeutic efficacy [38]. Their ability to reflect dynamic changes in tau pathology makes them invaluable tools in the development and evaluation of disease-modifying therapies targeting amyloid and tau [38].

Despite the considerable promise of plasma p-Tau isoforms, several challenges remain before their widespread clinical adoption. One major issue is inter-assay variability, which can arise from differences in assay platforms, reagents, and protocols, complicating comparisons across studies and laboratories [48]. Additionally, there is a lack of universally accepted standardized cut-off values, which limits clinical interpretability and decision-making [48]. Another critical limitation is the underrepresentation of diverse populations in biomarker validation studies; most research to date has focused on relatively homogeneous cohorts [49], often lacking sufficient inclusion of non-white and underserved groups. This gap raises concerns about the generalizability and equity of biomarker-based diagnostics.

Addressing these challenges requires ongoing international efforts to harmonize assay methodologies, establish robust clinical validation across diverse populations, and develop standardized guidelines for interpretation [50]. Such initiatives are essential to fully harness the potential of plasma p-Tau biomarkers in advancing precision medicine approaches for AD, ultimately enabling earlier diagnosis, improved patient stratification, and more effective therapeutic monitoring.

### 2.3. Neurofilament Light Chain

Neurofilament light chain is a structural cytoskeletal protein predominantly found in large, myelinated axons, where it supports axonal caliber and conduction velocity [51]. Upon neuronal injury, NfL is released into the interstitial fluid and subsequently diffuses into cerebrospinal fluid and blood [52]. Elevated plasma NfL levels serve as a sensitive marker of neuroaxonal damage and neurodegeneration across a broad spectrum of neurological disorders [53,54,55].

In AD, plasma NfL concentrations progressively increase with disease severity and correlate with key indicators of neurodegeneration, including hippocampal atrophy and global brain volume loss on MRI, and cognitive decline [56,57]. Longitudinal studies have demonstrated that plasma NfL rises years before clinical symptoms appear, with evidence from familial AD cohorts indicating NfL elevations up to 10–15 years prior to the expected symptom onset [58]. Moreover, plasma NfL predicts conversion from MCI to AD dementia and correlates with both structural MRI changes and cognitive performance.

Despite its sensitivity, NfL lacks specificity for AD, as elevated levels are also observed in other central nervous system diseases, such as ALS, multiple sclerosis, vascular dementia, traumatic brain injury, and various neuroinflammatory conditions [59]. Additionally, factors like aging, vascular risk, and comorbidities can influence plasma NfL levels, further complicating interpretation. Therefore, NfL is most informative when combined with disease-specific biomarkers, such as β-amyloid and p-Tau, to improve diagnostic accuracy [28].

Hence, the dosage of plasmatic NfL levels is extensively used in various clinical scenarios, and is often combined with other biomarkers when the suspicion of a neurodegenerative disorder rises. Moreover, the utility of NfL in the management of AD patients extends beyond the diagnostic phase, since it has been proposed as a marker of therapeutic response in clinical trials [60,61]; serial plasma NfL measurements could in fact contribute to longitudinal monitoring of neurodegeneration over time.

Recent advances in mass spectrometry have further refined the characterization of NfL proteoforms in plasma, enhancing our understanding of its molecular complexity, and providing potential for improved biomarker development and performance [62]. However, challenges remain, including assay standardization, definition of robust clinical cut-offs, and validation across diverse populations [63].

### 2.4. Glial Fibrillary Acid Protein

Glial fibrillary acidic protein (GFAP) is an intermediate filament protein predominantly expressed by astrocytes that serves as a marker of astrogliosis and neuroinflammation, processes now recognized as early and critical components of AD pathophysiology [64]. Elevated plasma GFAP levels reflect astrocyte activation and correlate strongly with both amyloid burden, measured by PET imaging, and cognitive decline [65].

Emerging evidence suggests that GFAP becomes abnormal earlier than tau biomarkers, positioning it as a potential early indicator of AD-related pathology [66]. Studies have shown that plasma GFAP discriminates amyloid-positive from amyloid-negative individuals with a high sensitivity, particularly in asymptomatic or prodromal stages [67]; it has been demonstrated that GFAP outperforms neurofilament light chain and some tau biomarkers in distinguishing amyloid status.

Longitudinal data reveals that plasma GFAP levels rise up to a decade before the onset of cognitive impairment and remain elevated through disease progression [68]. It has been shown that GFAP levels can predict MCI-to-dementia conversion [67]. This early elevation likely reflects astrocytic responses to amyloid accumulation and compromised blood–brain barrier integrity, consistent with the neuropathological findings of increased GFAP expression around amyloid plaques and reactive astrocytosis observed in prodromal AD.

However, although brain-specific [67], GFAP is not AD-specific, since elevated levels are also observed in other neurological conditions, such as traumatic brain injury, multiple sclerosis, and stroke, and can also be influenced by systemic inflammation.

Therefore, it is advisable to include GFAP in multi-biomarker panels, alongside disease-specific biomarkers, in order to maximize its diagnostic and prognostic utility and interpret results through a more comprehensive perspective [28].

Recent meta-analyses and large cohort studies confirm the clinical potential of blood GFAP measurements to improve early diagnosis of AD and disease monitoring [23,67,69].

As neuroinflammation is increasingly recognized as a possible target of disease-modifying therapies, GFAP is also being evaluated as a marker of treatment-response in clinical trials, especially in those targeting glial activation [67].

An overview of the main aspects of plasma biomarker is available in Table 1.

### 2.5. Other Inflammatory Biomarkers

Neuroinflammation has emerged as a pivotal contributor to the pathogenesis of AD, intricately intertwined with the classical hallmarks of amyloid-β accumulation and tau pathology [3]. This inflammatory process involves a dynamic interplay between activated microglia, reactive astrocytes, and the release of a variety of pro-inflammatory cytokines and chemokines. Rather than being a mere bystander, neuroinflammation actively exacerbates neuronal damage and accelerates disease progression, creating a vicious cycle that amplifies amyloid and Tau pathology and contributes to synaptic dysfunction and neurodegeneration [4].

In recent years, several inflammatory biomarkers detectable in blood have been identified as potential indicators of neuroinflammatory response in AD [75]. Although these markers are generally nonspecific, and thus less suitable for standalone diagnosis, they hold considerable promise for monitoring disease progression and therapeutic response. Among these, YKL-40 (chitinase-3-like protein 1 or CHI3L1) is one of the most studied [76]. Primarily secreted by astrocytes and microglia, elevated levels of YKL-40 in both plasma and cerebrospinal fluid reflect glial activation and correlate strongly with tau pathology and cognitive decline. Importantly, YKL-40 levels tend to increase progressively along the AD continuum, making it a valuable candidate for staging disease severity and tracking neuroinflammatory activity over time [70].

Another key biomarker is soluble Triggering Receptor Expressed on Myeloid cells 2 (sTREM2), a fragment of the microglial surface receptor TREM2. sTREM2 levels in plasma and CSF rise notably during early symptomatic stages of AD and are thought to represent a compensatory microglial response to accumulating amyloid plaques [71]. This biomarker is particularly sensitive in the late preclinical phase, reflecting microglial activation before overt clinical symptoms manifest. Its dynamic changes offer insights into the evolving neuroimmune landscape of AD and provide a window into microglial function and dysfunction [72].

Pro-inflammatory cytokines, such as interleukin-6 (IL-6), tumor necrosis factor-alpha (TNF-α), and interleukin-1β (IL-1β) are frequently found at elevated levels in AD patients, indicative of both systemic and central nervous system inflammation [73]. However, their clinical utility as diagnostic markers is limited by considerable variability and notable lack of specificity, as these cytokines are elevated in numerous other inflammatory and infectious conditions.

The advent of multiplex proteomic platforms, such as Olink and SomaScan [74], has revolutionized the ability to quantify dozens to hundreds of inflammatory proteins simultaneously in plasma samples. These high-throughput technologies have uncovered distinct inflammatory signatures associated with different stages of AD and varying cognitive trajectories. While individual inflammatory markers alone lack diagnostic specificity, their combination into multi-analyte panels alongside core AD biomarkers substantially enhances their utility; indeed, this integrative approach supports earlier detection and more precise monitoring of disease progression [28].

Longitudinal studies have further demonstrated that plasma levels of YKL-40, and sTREM2 increase progressively from preclinical to symptomatic stages of AD [77]. Beyond their diagnostic and prognostic value, these neuroinflammatory biomarkers hold significant promise in the context of clinical trials, particularly those evaluating anti-inflammatory or glial-targeting therapies. They offer minimally invasive tools to monitor therapeutic response and disease progression, complementing imaging biomarkers, such as translocator protein (TSPO) and monoamine oxidase B (MAO-B) PET tracers, which visualize glial activation in vivo [3]. This multimodal approach enhances the ability to assess target engagement and treatment efficacy in real time.

Despite their potential, the clinical application of neuroinflammatory blood biomarkers faces challenges, primarily due to their nonspecific nature [77]. Given that elevated levels of YKL-40, sTREM2 and GFAP are also observed in other neurological disorders, including stroke, multiple sclerosis, and traumatic brain injury, their measurement should be integrated into a broader panel of biomarkers, in order to avoid misinterpretation.

### 2.6. Other Emerging Biomarkers

Beyond the more-established biomarkers of Aβ, tau, neurodegeneration and neuroinflammation, research has increasingly focused on a diverse array of novel plasma biomarkers that capture additional, critical aspects of AD pathophysiology. These emerging markers reflect processes such as synaptic loss, oxidative stress, metabolic dysregulation, and proteostasis impairment, all of which contribute to the complex biological landscape of AD.

Synaptic dysfunction represents one of the earliest pathological events in AD, often preceding overt neuronal loss and clinical symptoms. Plasma levels of synaptic proteins, including neurogranin, synaptosomal-associated protein 25 (SNAP-25), and synaptotagmin, have been shown to correlate with synaptic integrity and cognitive performance [78,79,80]. These proteins serve as sensitive indicators of synaptic health, providing insights into brain functionality that go beyond structural imaging or gross neurodegeneration. As such, they are currently emerging as promising biomarkers for early detection and monitoring of disease progression.

Oxidative stress is another hallmark of AD pathology, driven largely by mitochondrial dysfunction and the accumulation of reactive oxygen species [4]. This leads to widespread cellular damage, including lipid peroxidation, DNA oxidation, and protein modification. Several oxidative stress markers, such as 8-hydroxy-2′-deoxyguanosine (8-OHdG), malondialdehyde (MDA), and F2-isoprostanes, have been found elevated in the plasma of AD patients [81,82,83,84]. Despite their biological relevance, challenges related to assay standardization, reproducibility, and sensitivity have limited their translation into routine clinical use, underscoring the need for further methodological refinement.

Metabolomic and lipidomic profiling have revealed consistent alterations in amino acid and lipid metabolism in individuals with AD [85]. Shifts in plasma levels of phosphatidylcholine, ceramides, and acylcarnitines have been documented, distinguishing AD patients from cognitively normal controls, and potentially reflecting early metabolic disturbances that precede clinical decline [86]. These metabolic signatures not only enhance diagnostic accuracy, but also provide valuable insights into disease mechanisms, including mitochondrial dysfunction and altered lipid homeostasis, which may serve as therapeutic targets [87].

Proteostasis dysfunction, characterized by impaired protein folding, trafficking, and degradation, is another critical feature of AD. Blood-derived neuron-specific extracellular vesicles, or exosomes, offer a unique “liquid brain biopsy” by carrying neuronal proteins, such as Aβ, phosphorylated Tau, and synaptic markers [88]. Exosomal tau phosphorylated at multiple sites has demonstrated high diagnostic accuracy and shows potential for early detection and prediction of cognitive decline, making exosome analysis a particularly exciting frontier in biomarker research [89].

Moreover, recent advances in proteomic technologies have facilitated the identification of novel plasma biomarkers associated with AD pathology across diverse populations. For instance, brain-derived neurotrophic factor (BDNF), a key regulator of neuronal survival and plasticity, has shown strong associations with neurodegeneration [90], while the above-mentioned synaptic proteins exhibit correlations with APOE genotype, highlighting genetic influences on biomarker expression.

However, numerous inflammatory and vascular-related proteins have also been linked to demographic factors, such as age, race, and sex, emphasizing the need to consider population diversity in biomarker research and interpretation.

Innovative experimental approaches have also contributed to new biomarker discovery, such as proteins implicated in endothelial cell migration and vascular remodeling, suggesting new pathways involved in cerebrovascular dysfunction in AD and offering potential targets for therapeutic intervention [91].

Although these emerging biomarkers hold great promise, they largely remain in the discovery or early validation stages. To transition from research to clinical application, extensive longitudinal studies in multi-ethnic cohorts are essential to establish their robustness, reproducibility, and generalizability [92]. Moreover, assay standardization and integration with established AD biomarkers will be critical to ensure accuracy and clinical utility. The future of AD diagnostics is likely to involve sophisticated multi-analyte panels that combine these novel markers with classical biomarkers, leveraging machine learning and systems biology approaches to enhance early detection, prognostication, and personalized treatment strategies [93].

Table 2 summarizes the principal characteristics of emerging blood biomarkers, focusing on those related to neuroinflammation, synaptic impairment, oxidative stress and metabolic alterations.

## 3. Analytical Techniques

Technological advancements have played a crucial role in enabling the reliable detection and quantification of blood biomarkers for AD, facilitating both research and clinical applications. Among the most prominent platforms is Simoa technology, which employs digital immunoassays capable of isolating and quantifying individual protein molecules with femtomolar sensitivity [95]. This ultra-sensitive approach has been widely adopted for measuring plasma biomarkers, such as p-Tau isoforms, NfL, and GFAP. For example, Simoa assays for p-Tau217 have consistently demonstrated diagnostic accuracies exceeding 90% in distinguishing AD patients from controls across diverse cohorts, highlighting their robustness and reproducibility [96]. However, the high cost and requirement for specialized instrumentation may limit their accessibility in some clinical settings, particularly those with limited resources.

Lumipulse technology represents a significant advance, offering fully automated immunoassays for the measurement of key plasma biomarkers including p-Tau181, p-Tau217, and Aβ42/40 [33,49,50]. Lumipulse assays are notable for their ease of use, high throughput, and integration into routine clinical laboratory workflows, making them highly attractive for large-scale screening and longitudinal monitoring. While not as ultrasensitive as Simoa, Lumipulse assays provide a pragmatic balance between sensitivity, specificity, and accessibility, thus enhancing the feasibility of implementing blood-based biomarkers in diverse clinical environments [97].

Another sophisticated technique is IP-MS, which combines antibody-based enrichment with the precision of mass spectrometry to achieve highly specific quantification of protein isoforms and post-translational modifications [98]. This method is particularly valuable for measuring plasma Aβ42/40 ratios, with studies showing strong correlations between IP-MS results and amyloid PET imaging, as well as cerebrospinal fluid Aβ levels. Despite its analytical strengths, IP-MS demands specialized expertise and infrastructure, which may restrict its widespread adoption in routine clinical practice [99].

Electrochemiluminescence immunoassays (ECLIA) offer multiplexed quantification of biomarkers, including total tau and Aβ isoforms [100]. While generally less sensitive than Simoa, ECLIA provides good reproducibility and scalability, making it well-suited for research applications and increasingly adaptable to clinical workflows. Its multiplexing capability allows simultaneous measurement of multiple analytes, enhancing efficiency and data richness isoforms [98].

Recent progress in fully automated immunoassay platforms has further advanced the field by enabling high-throughput, standardized measurement of plasma biomarkers. These technologies combine performance comparable to advanced platforms with ease of implementation in routine clinical laboratories [101]. Automation is critical for scaling up screening efforts and longitudinal monitoring of AD in real-world settings, addressing the need for accessibility and reproducibility.

Collectively, these technological innovations provide a comprehensive toolkit for advancing blood-based biomarker research and clinical application in AD. Each platform offers distinct advantages and limitations, and their complementary use in a collective international setting is likely to enhance diagnostic accuracy, prognostic capability, and therapeutic monitoring [101]. Continued development and integration of these technologies, alongside efforts to improve accessibility and standardization, will be essential to fully realize the potential of blood biomarkers in AD care [102].

## 4. Context of Use, Challenges and Limitations of Blood Biomarkers

Blood-based biomarkers are poised to fundamentally reshape the management and understanding of AD, offering new opportunities for early detection, risk stratification, and longitudinal monitoring across the entire disease continuum. Their accessibility and scalability make them especially well-suited in identifying individuals at preclinical risk, when interventions could be most impactful intervention [5,103].

This potential for early detection hints a significant transformation in clinical practice. The employment of blood tests could reduce the need for PET imaging and lumbar punctures, thereby allowing the avoidance of costly exams and invasive procedures. The enhanced feasibility of large-scale screening could lead to a remodulation of the diagnostic flowchart, and to an advantageous distribution of resources among centers with increasing levels of expertise in the field of neurodegenerative disorders [96].

**Figure 1:**
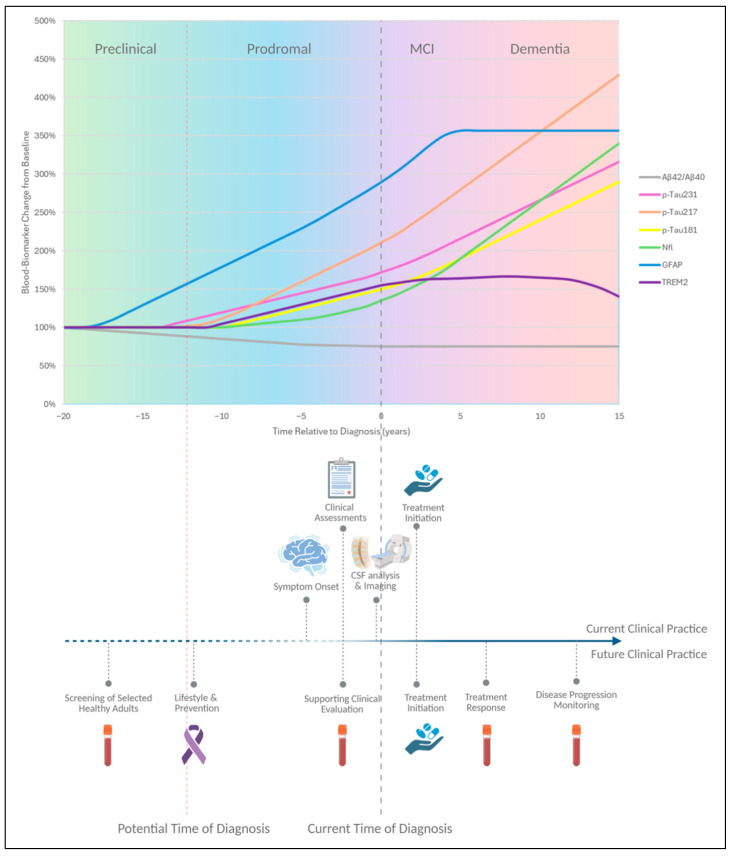
Temporal trajectories of blood biomarkers across the Alzheimer’s disease continuum, from the preclinical phase to dementia. Early changes precede symptom onset by many years, while subsequent increases reflect progression of pathology, neurodegeneration, and glial activation. The lower panel contrasts current reliance on CSF and imaging with future perspectives where blood biomarkers will support early detection, stratification, therapeutic monitoring and longitudinal follow-up. This figure was created with Biorender.com.

Figure 1 summarizes current evidence on blood biomarker dynamics across the AD continuum and outlines both established and potential contexts of clinical application.

However, the real-world implementation of blood biomarkers demands careful critical appraisal [104,105]. The adoption of a two cut-off approach, as recommended by the Global CEO Initiative on Alzheimer’s Disease, underscores both the strengths and inherent challenges of current assays: while this strategy improves diagnostic precision by establishing clear thresholds and an intermediate “gray zone,” it also exposes persistent issues related to assay variability and the lack of universal standards, which complicate consistent interpretation across different clinical and research settings [106,107,108].

Furthermore, the introduction of a “gray zone” mandates the careful arrangement of a third, specific, integrated care protocol dedicated to those subjects who result neither “frankly positive” nor “frankly negative” to blood biomarker testing. Such a new category of individuals certainly opens new horizons in the understanding of AD spectrum, but also entails ethical ramifications, imposing major changes in the healthcare system, so to provide every patient with the most adequate assistance and support.

Moreover, despite recent technological advances, including the development of automated, high-throughput platforms, widespread integration into routine clinical workflows remains hampered by infrastructure requirements and cost, particularly in resource-limited environments where access to traditional diagnostics is already constrained [101].

Longitudinal measurements of biomarkers like NfL and GFAP offer valuable insights into neurodegeneration and disease progression, but their prognostic utility is limited by inter-individual variability and the absence of standardized thresholds [63]. Similarly, while these markers have advanced our understanding of AD heterogeneity and enabled more nuanced patient stratification, biological and technical variability continue to challenge the reproducibility of subtyping efforts.

The regulatory landscape is rapidly evolving, as proven by the recent FDA clearance of Lumipulse plasma pTau217/Aβ42 ratio. This ratio has shown reliability in predicting the presence of amyloid plaques in cognitively impaired patients referring to specialized Memory Centers [109]. The ratio undoubtedly draws its power from its capacity of encompassing both the amyloid-related and the tau-related pathology, and is therefore preferable to the classical mono-pathology biomarkers. However, the FDA reaffirms the importance of interpreting all results in light of clinical history.

Moreover, the ongoing development of clinical practice guidelines by the Alzheimer’s Association reflects both the promise and the need for evidence-based recommendations to guide who should be tested and when [104].

This regulatory progress, alongside rapid scientific innovation, stresses the importance of balancing enthusiasm for technological advances with rigorous validation and critical interpretation [110].

Ultimately, blood-based biomarkers represent a powerful and evolving toolkit for advancing AD care and research, but their ethical and effective widespread use requires ongoing attention to issues of analytical rigor, population diversity, and clinical correlation [92]. Only by integrating technological innovation, robust validation, and equitable access can the full potential of blood biomarkers be realized in transforming the landscape of AD [104,105].

While blood-based biomarkers hold promise for transforming the landscape of AD diagnosis, prognosis, and treatment, several critical challenges must be addressed to fully realize their clinical potential. One of the most significant obstacles is the lack of standardization and harmonization across assays and laboratories. Variability in pre-analytical procedures [111], such as sample collection and processing, along with differences in assay calibration, reagents, and interpretation thresholds, undermines the comparability of biomarker measurements obtained from different studies and platforms [112]. This inter-laboratory variability complicates the establishment of universally applicable cut-off values, hindering clinical adoption and limiting the ability to pool data across studies for meta-analyses or large-scale validation efforts. To overcome these issues, international consortia and regulatory bodies are actively engaged in developing harmonized guidelines, reference materials, and standardized workflows [74].

Another critical challenge revolves around population diversity and generalizability. The vast majority of existing biomarker research has been conducted in relatively homogeneous, well-characterized cohorts, often lacking adequate representation of diverse ethnic, racial, and socioeconomic groups [92]. This representation raises concerns about the generalizability of findings and the potential for disparities in diagnostic accuracy across different populations [113,114]. It is imperative to conduct validation studies in more diverse cohorts, including individuals with varying comorbidities that may influence biomarker levels. Such studies are essential to ensure that blood biomarkers perform consistently and fairly across different demographic and clinical contexts, promoting equitable access to biomarker-based diagnostics and personalized treatment strategies for all individuals at risk of AD [115].

In addition to technical and scientific considerations, the deployment of blood biomarkers, particularly for early detection and screening of asymptomatic individuals, introduces complex ethical and practical considerations [116]. Disclosure of biomarker results indicating increased AD risk may cause psychological distress, anxiety, or stigma, particularly in the absence of widely effective preventive or curative treatments [117]. Moreover, there are potential implications for employment, and social discrimination, underscoring the need for careful consideration of the societal impact of biomarker-based diagnostics [116].

To address these ethical concerns, it is essential to develop clear guidelines along with counseling practices that ensure that individuals are fully informed about the potential benefits and risks of biomarker testing, empowered to make autonomous decisions regarding their healthcare, and protected from potential harms associated with biomarker disclosure [117].

Finally, from an organizational point of view, the introduction of blood biomarkers in an operating center imposes the renewal of clinical practice and a reshaping of protocols. Such a challenging transformation is not effortless, and likely requires a transition period.

## 5. Discussion and Conclusions

Blood-based biomarkers are catalyzing a transformative shift in the diagnosis, monitoring, and management of Alzheimer’s disease. Their minimally invasive nature, scalability, and steadily improving diagnostic accuracy offer substantial advantages over traditional diagnostic tools, such as CSF analysis and PET imaging. These biomarkers hold promise for enabling earlier detection of AD, facilitating more precise prognostication, and streamlining clinical trial designs, thereby accelerating the development of effective therapies [118].

A comprehensive, multi-modal biomarker strategy that merges plasma Aβ42/40, p-Tau isoforms, NfL, and GFAP has demonstrated remarkable diagnostic accuracy, compensating for the weaknesses of single biomarker measurements [28].

However, these exciting advances in the field of blood-based biomarkers do face a challenging and complex translation into routine clinical practice [115]. Indeed, their widespread implementation necessitates continued efforts toward assay standardization, rigorous validation across diverse populations, regulatory approval, and establishment of ethical frameworks to ensure responsible and equitable use [116]. Furthermore, guidelines approved by the community of experts must be translated in the real-world setting, considering the vast heterogeneity of cultural and clinical scenarios, and the peculiar features of each single reality.

Addressing these challenges will be critical to realizing the full potential of blood biomarkers in transforming AD care.

As novel anti-amyloid and other disease-modifying therapies continue to evolve [118], accessible and reliable blood biomarkers will become indispensable tools for identifying appropriate candidates for treatment, monitoring therapeutic efficacy in real time, and refining clinical decision-making to optimize patient outcomes [119]. To support this, international collaborations must prioritize harmonizing pre-analytical procedures, assay platforms, and interpretation criteria to ensure consistency and comparability across clinical and research settings [111]. Expanding research to include ethnically, racially, and socioeconomically diverse, community-based populations will enhance the generalizability of findings and promote equity in diagnosis and care. Ethical oversight frameworks addressing informed consent, risk communication, and data privacy are equally vital, particularly as biomarker testing moves toward earlier, pre-symptomatic diagnosis [117]. Finally, integrating blood biomarkers into comprehensive screening, diagnostic, and therapeutic algorithms across both specialist and primary care environments will be crucial for maximizing their clinical impact.

The field has progressed rapidly, moving from conceptual research to tangible clinical application. Looking ahead, the integration of these biomarkers into multidimensional diagnostic models, combining clinical assessments, genetic information, cognitive testing, and lifestyle factors, will be critical [120].

Key areas for future research include conducting longitudinal studies across ethnically and clinically diverse cohorts to capture the full spectrum of AD heterogeneity, developing multi-biomarker panels to enable disease subtyping and more accurate prognosis, validating biomarkers as surrogate endpoints in therapeutic trials, implementing blood biomarker testing in real-world primary care settings, and establishing robust ethical frameworks for biomarker disclosure and clinical decision-making [119].

Blood-based biomarkers promise to revolutionize Alzheimer’s care by shifting it from a late-stage model to a proactive and timely personalized approach. This transformation will enable the early identification of individuals at risk, guide targeted therapies tailored to individual disease biology, and facilitate continuous, minimally burdensome monitoring of disease progression, thereby improving outcomes for patients and alleviating strain on healthcare systems worldwide.

## 6. Search Strategy

For this narrative review on blood-based biomarkers in Alzheimer’s disease, a comprehensive literature search was conducted to capture the most recent and relevant advances in the field. Databases, including PubMed, Scopus and Web of Science, were queried using a combination of keywords and MeSH terms related to Alzheimer’s disease, blood biomarkers and diagnostic technologies. The search prioritized original research articles, clinical trials, systematic reviews and meta-analyses published up to early 2025. Emphasis was placed on studies reporting biomarker discovery, validation, clinical applications and technological advancements. Selection criteria favored studies that elucidate the biological relevance, diagnostic accuracy, and translational potential of blood biomarkers across the AD continuum. Articles addressing challenges, ethical considerations and future directions were also included to provide a holistic perspective. Reference lists of key publications were hand-searched to identify additional pertinent studies.

## Figures and Tables

**Table 1:** Overview of established blood biomarkers in Alzheimer’s disease.

Blood Biomarker	Biological Role	Detection Method	Diagnostic Utility	Limitations	References
Aβ42/Aβ40	Reflects amyloid plaque formation and deposition	Simoa, IP-MS, Lumipulse	Early detection of amyloid pathology; correlates with amyloid PET; useful in preclinical stages	Low plasma abundance; peripheral metabolism confounds levels; assay variability across platforms	[24,70]
p-Tau181, p-Tau217, p-Tau231	Reflect amyloid and tau pathology	Simoa, IP-MS, Lumipulse	Specificity for AD; distinguishes AD from other dementias; early and prodromal stage detection; prognostic for progression	Inter-assay variability; need for standardized cut-offs; emerging data for p-Tau231	[34,35,71]
NfL	Marker of axonal injury and neurodegeneration	Simoa, IP-MS, Lumipulse	Tracks disease progression; prognostic value for cognitive decline	Not specific to AD; elevated in other neurodegenerative diseases and with aging; influenced by comorbidities	[72,73]
GFAP	Astrocytic activation and gliosis	Simoa, IP-MS, Lumipulse	Early marker of astroglial activation; correlates with amyloid pathology; predictive of cognitive decline	Elevated in other neurological conditions; assay standardization needed	[74]

**Table 2:** Emerging Blood Biomarkers in Alzheimer’s Disease: Biological Roles, Detection, and Clinical Relevance.

Biomarker	Biological Role	Detection Method(s)	Current Evidence	Limitations	References
Neuro-inflammation YKL-40, sTREM2, IL-6, TNF-α, IL-1β	Markers of astrocyte and microglial activation; mediators of systemic and central inflammation	ELISA, Simoa Metabolomic platforms (Olink, SomaScan)	Reflect neuroinflammation; correlate with Tau pathology and cognitive decline; track disease progression	Moderate to low specificity; assay variability; influenced by systemic conditions	[70,71]
Synaptic Markers Neurogranin, SNAP-25	Reflect synaptic integrity and dysfunction	ELISA, Simoa	Early indicators of synaptic loss; correlate with cognitive impairment	Limited plasma validation; variable assay sensitivity; less studied in blood	[88]
Oxidative Stress Markers 8-OHdG, Malondialdehyde, F2-isoprostanes	Indicators of oxidative DNA and lipid damage	ELISA, Mass Spectrometry	Reflect oxidative stress contributing to AD pathology; may indicate progression	Assay variability; low specificity; influenced by systemic oxidative stress	[82]
Metabolomic Markers Phosphatidyl-choline, Sphingomyelin	Reflect lipid metabolism and membrane integrity	Mass Spectrometry, Metabolomic platforms (Olink, SomaScan)	Altered lipid profiles in AD; potential early markers; improve diagnostic panels	High inter-individual variability; best used in multi-marker panels	[94]

This table summarizes key Alzheimer’s disease biomarkers. Neuroinflammatory markers like YKL-40, sTREM2, and cytokines (IL-6, TNF-α, IL-1β) rise early and track progression but have moderate specificity and variability. Synaptic markers such as neurogranin and SNAP-25 reflect early synaptic loss, though plasma validation is limited. Oxidative stress markers (8-OHdG, MDA, F2-isoprostanes) indicate damage but lack disease specificity. Metabolomic markers, including changes in phosphatidylcholine and sphingomyelin, reflect lipid metabolism disruption and are best used in multi-analyte panels due to variability.
